# 

**Published:** 1873-06

**Authors:** 


Vol. XI. JUNE, 1873. No. 3.

### ATLANTA MEDICAL AND SURGICAL JOURNAL

A MONTHLY DEVOTED TO

### THE MEDICAL SCIENCES.

EDITORS:

JOSEPH P. LOGAN, M.D.,

AND

W. F. WESTMORELAND, M.D.,

*Professor of the Principles and Practice of Surgery in Atlanta Medical College.*

CORRESPONDING EDITOR:

ROBERT BATTEY, M.D., Rome, Georgia.

ASSOCIATE EDITORS:

CH. RAUSCHENBERG, M.D.

J. G. WESTMORELAND, M.D. | V. H. TALIAFERRO, M.D.

*PAX ET SCIENTIA, SED VERITAS SINE TIMORE.*

ATLANTA, GEORGIA:

Herald Publishing Company's Steam Press.

1873.

